# Cancer Screening Rate and Related Factors in the Japanese Child-Rearing Generation

**DOI:** 10.3390/healthcare10030508

**Published:** 2022-03-10

**Authors:** Mutsumi Okayama, Tomo Nagaoka, Koshu Sugisaki

**Affiliations:** 1 Department of Health and Welfare, Graduate School of Niigata University of Health and Welfare, 1398 Shimami-cho, Kita-Ku, Niigata 950-3198, Japan; okayama@jumonji-u.ac.jp (M.O.); tm-nagaoka@juntendo.ac.jp (T.N.); 2 Department of Psychology, Faculty of Education and Humanities, Jumonji University, 2-1-28 Sugasawa, Niiza 352-8510, Japan; 3 Department of Sports Science, Juntendo University, 1-1 Hiraka-gakuendai, Inzai 270-1695, Japan; 4 Department of Health and Sports, Niigata University of Health and Welfare, 1398 Shimami-cho, Kita-Ku, Niigata 950-3198, Japan

**Keywords:** parents, income, education, cancer-screening, child-rearing

## Abstract

In Japan, although the incidence of cancer is increasing, the cancer screening rate is low compared to that in other countries. This study aimed to evaluate the factors associated with cancer screening behavior in Japanese men and women of child-rearing age. The survey was conducted among 2410 child-rearing adults from a countrywide database in August 2018. Among the respondents, there were 1381 (57.3%) who had been screened for cancer and 1029 (42.7%) who had not been screened. When stratified by sex, 503 (40.9%) men and 878 (74.3%) women had been screened for cancer, and education, income, and family history were associated with cancer screening. Among the men, where they lived, age, and family history were associated with cancer screening. In women, annual income was associated with stomach, colon, breast, and uterine cancer screening. In addition, uterine cancer screening was related to the women’s educational level. Our results suggest a need to improve the cancer screening rate among the child-rearing generation, especially for those with limited education and low income.

## 1. Introduction

Cancer is the first or second-leading cause of death before the age of 70 years in 91 out of 172 countries. Cancer incidence and mortality are rapidly increasing worldwide [1]. An estimated 18.1 million new cancer cases and 9.6 million cancer deaths globally were projected in 2018 [1]. In Japan, the incidence of cancer is increasing, with one million new cancer cases and 0.38 million cancer deaths projected in 2020 [2]. Further, the prevalence of large intestine, stomach, lung, prostate, and breast cancers are increasing, in that order [2]. Moreover, the risk of cancer occurrence is high among men aged 60 years and above [3]. Although the prevalence of cancer increases for both men and women above the age of 50, it is higher in women than in men for persons in their 20s and early 50s.

In Japan, employers are obligated to provide health checkups to their workers once a year, and workers are required to take them. At that time, cancer screenings are available to company employees and civil servants at the time of their health checkups. In addition, under the Japanese health insurance system, all citizens, even those who are unemployed, can undergo cancer screening at the municipality at a low cost if they are of the recommended age. The age for screening is ≥20 years for cervical cancer; ≥40 years for lung, colorectal, and breast cancers; and ≥50 years for stomach cancer [4]. In recent years, the age at first marriage has increased for both men and women, subsequently increasing the childbearing age as well. Therefore, it is highly feasible that the children of cancer patients are minors.

The cancer survival rate has been increasing due to early detection and treatment. Cancer screening is indispensable for early detection. The breast cancer screening uptake rate is >80% in the United States and >70% in Italy and the Netherlands [5]. Although the screening rate has improved in Japan, it is 44% for breast cancer screening. In addition, the cancer screening rate among women in Japan is often lower than that among men [6]. In Japan, there are various factors associated with cancer screening behavior. Age, educational level, income, cost of screening, and a family history of cancer have been found to influence cancer screening behavior [7,8]. However, factors influencing cancer screening behavior in adults of child-rearing age have not been analyzed. Therefore, this study aimed to evaluate the factors associated with cancer screening behavior in Japanese men and women of child-rearing age.

## 2. Materials and Methods

**Figure 1:**
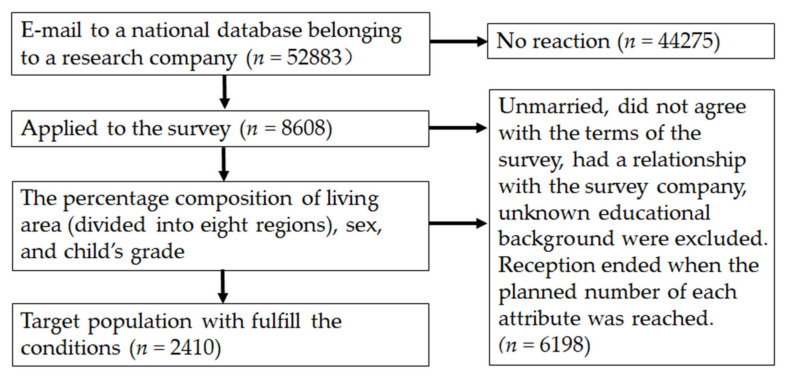
Participant flow.

The study population was selected from a national database belonging to a research company. The survey targets (52,883) registered with the survey contractor were notified by e-mail. Although 8608 people applied to the survey company, the survey responses in cases of persons who were unmarried, did not agree with the terms of the survey, or had a relationship with the survey company and unknown educational background were excluded. The responses from a region/grade level were discontinued when the expected collection targets were reached in that region/grade. The study population was divided into eight regions (Hokkaido and Tohoku, Kanto, Hokuriku, Chubu, Kansai, Chugoku, Shikoku, and Kyushu) of Japan, and the percentage composition of living area, sex, and child’s grade was calculated. The number of participants who fulfilled the conditions was 2410 (Figure 1). The survey was conducted in August 2018 on the internet, and the participants were required to respond immediately. The survey questions included sex (male or female), living area, number of children, children’s grade, educational background, household income, history of cancer screening, types of cancer screenings, and family experience with cancer, each of which was selected from a list of options.

In Japan, the recommended age for major cancer screenings is over 40 years old. (Uterine cancer screening is recommended for people over 20 years old.) Therefore, we analyzed the data with 40 year olds and 20 year olds as the cutoff points.

A logistic regression analysis (forced entry method) was performed on age, educational level, household income, and family history, and the odds ratios and 95% confidence intervals (CIs) were calculated. All multivariate analyses were adjusted for the living area. All analyses were performed using IBM SPSS 26.0 Statistics for Macintosh, version 26 (IBM Corp., Armonk, NY, USA), and the level of significance was set at *p* < 0.05.

### Compliance with Ethical Standards

We received approval from the ethics committee of Niigata University of Health and Welfare before conducting the study. Prior to conducting the web survey, we obtained informed consent online for all the participants. At the time of the survey, it was communicated in the letter of request that the individuals would not be identified and that their freedom to participate was guaranteed, and the survey was carried out with their consent. Participants who agreed to participate in this survey voluntarily responded to the questionnaire and collected information anonymously without revealing the identity of individual participants.

## 3. Results

### 3.1. Demographic Characteristics of Both Sexes

The demographic characteristics of the survey participants are presented in Table 1.

### 3.2. Cancer Screening Experience

There were 1381 (57.3%) participants who had been screened for cancer and 1029 (42.7%) who had not been screened. When stratified by sex, 503 (40.9%) men and 878 (74.3%) women had been screened for cancer. Men who had been screened for cancer were more likely to have an annual income ≥ 8,000,000 yen (42.4%) and to have graduated from university or higher education (73.3%). Women were more likely to have been diagnosed with breast and uterine cancer screening (70.2% and 66.3%, respectively) (Table 2).

### 3.3. Factors Associated with Cancer Screening Experience

The factors associated with a cancer screening experience are presented in Table 3. The odds of screening women compared to screening men aged >40 years increased by 5.31 (95% CI, 4.24–6.65) (*p* < 0.001). The odds of screening for cancer increased with an income ≥ 8,000,000 yen compared to those with an income < 4,000,000 yen by 1.79 (95% CI, 1.26–2.54) (*p* = 0.001). The odds of having a university or graduate school education were 1.36 (95% CI, 1.04–1.78) (*p* = 0.024), and the odds of having a family member with cancer were 1.69 (95% CI, 1.38–2.07) (*p* < 0.001) (Table 3).

### 3.4. Associations with Screening for Different Cancers

#### 3.4.1. Men

The factors associated with site-specific cancer screening for men are shown in Table 4. For lung cancer, the odds of having a family member with lung cancer compared to those without cancer were 1.43 (95% CI, 1.06–1.93) (*p* = 0.019). The odds of being in the 50s and 60s age groups compared to those of being in the 40s age group were 1.89 (95% CI, 1.41–2.53) (*p* < 0.001) and 2.54 (95% CI, 1.25–5.14) (*p* = 0.010), respectively.

For stomach cancer, the odds of having an income ≥ 800,000 yen compared to those with an income < 400,000 yen were 1.71 (95% CI, 1.02–2.85) (*p* = 0.041). The odds of having a family member with stomach cancer compared to those without cancer were 1.57 (95% CI, 1.20–2.07) (*p* = 0.001). The odds of being in the 50s and 60s age groups compared to those in the 40s age group were 1.66 (95% CI, 1.26–2.19) (*p* < 0.001) and 2.72 (95% CI, 1.37–5.38) (*p* = 0.004), respectively.

For colorectal cancer, the odds of having a family member with colorectal cancer compared to those without cancer were 1.43 (95% CI, 1.09–1.88) (*p* = 0.011). The odds of being in the 60s age group compared to those in the 40s age group were 2.33 (95% CI, 1.18–4.59) (*p* = 0.015).

#### 3.4.2. Women

The factors associated with site-specific cancer screening in women are shown in Table 5. For lung cancer, the odds of having a family member with lung cancer compared to those without lung cancer were 1.53 (95% CI, 1.06–2.20) (*p* = 0.024).

For stomach cancer, the odds of having an income of 4,000,000–6,000,000 yen, 6,000,000–8,000,000 yen, or ≥8,000,000 yen compared to those with an income under 4,000,000 yen were 2.33 (95% CI, 1.34–4.08) (*p* = 0.003), 2.57 (95% CI, 1.45–4.56) (*p* = 0.001), and 2.35 (95% CI, 1.32–4.16) (*p* = 0.004), respectively. The odds of having a family member with stomach cancer compared to those families without cancer were 1.70 (95% CI, 1.23–2.34) (*p* = 0.001). The odds of being in the 50s age group compared to those in the 40s age group were 1.57 (95% CI, 1.02–2.41) (*p* = 0.041).

For colorectal cancer, the odds of having an income of 4,000,000–6,000,000 yen, 6,000,000–8,000,000 yen, or ≥8,000,000 compared to those with an income < 4,000,000 yen were 1.92 (95% CI, 1.16–3.20) (*p* = 0.012), 1.95 (95% CI, 1.15–3.31) (*p* = 0.014), and 2.36 (95% CI, 1.40–4.00) (*p* = 0.001), respectively. The odds of having a family member with colorectal cancer compared to those without such cancer were 1.77 (95% CI, 1.30–2.41) (*p* = 0.001).

For breast cancer, the odds of having an income of ≥800,000 yen compared to those with an income < 400,000 yen were 2.38 (95% CI, 1.43–3.95) (*p* = 0.001). The odds of having a family member with breast cancer compared to families without such cancer were 1.69 (95% CI, 1.24–2.30) (*p* = 0.001).

For uterine cancer, the odds of having an income of ≥800,000 yen compared to those with an income <400,000 yen were 1.79 (95% CI, 1.18–2.70) (*p* = 0.006) (Table 6). The odds of screening if one reported university or graduate school education compared to those screening that reported junior high or high school education were 1.50 (95% CI, 1.08–2.07) (*p* = 0.014). The odds of having a family member with uterine cancer compared to those without such cancer were 1.69 (95% CI, 1.31–2.18) (*p* < 0.001). The odds of being in the 30s, 40s, or 50s age groups compared to those in the 20s age group were 3.00 (95% CI, 1.09–8.31) (*p* = 0.034), 4.56 (95% CI, 1.68–12.47) (*p* = 0.003), and 3.58 (95% CI, 1.22–10.48) (*p* = 0.020), respectively.

## 4. Discussion

Various factors, including age, educational level, income, and a family history of cancer, have been reported to be associated with cancer screening [7,9,10,11]. However, these factors have only been examined for site-specific cancers. In the present study, in addition to the site-specific examination, the factors associated with cancer screening were also examined by sex. In the child-rearing generation, the subject of this study, the overall cancer screening rate, was significantly higher for women than for men. This is presumably because the screening rates for breast and uterine cancers among women are higher than for other cancers.

Similar to trends reported in previous studies, the screening rates for stomach and lung cancers were higher in men than in women [8]. However, unlike previous surveys, the colorectal cancer screening uptake rate in this study was higher among women, and the difference was due to the timing of the survey.

The percentage of participants with university or graduate school education was higher than that of those with junior or senior high school education. This is consistent with the results of previous studies, which showed the same trend for all cancer screenings [7]. This could be due to a relationship between higher education and more information about health status [12].

In this study, having a family member who had suffered from cancer was associated with cancer screening for all cancers: stomach, lung, colon, breast, and uterine. This result was similar to a previous study that examined the factors associated with uterine cancer screening among female workers [13]. Although the previous study was conducted on children, those who have parents or relatives with cancer have a better understanding about cancer; however, they also have stronger negative feelings about the disease [14]. Although this study targeted adults, it can be inferred that the presence of a cancer patient in the family is likely to increase the level of understanding about cancer and may promote intentions to undergo cancer screening. A previous study [8] also reported that the higher the level of concern about cancer, the more likely it is to result in cancer screening. It is presumed that the presence of a history of cancer in someone close, helps one understand the importance of screening, leading one to undergo screening rather than avoiding it, since some cancers, such as breast, colorectal, and ovarian cancers, have a genetic component [15].

The cancer screening rates in this study are low compared to those in other countries: 25.2%, 33.5%, and 31.2% for lung, stomach, and colorectal cancer, respectively [16]. Although a high percentage of the subjects in this study were in their 40s, the results suggest that the opportunity for cancer screening does not always lead to uptake.

In other countries, it has been reported that high levels of psychological distress, including fear and anxiety regarding cancer, are often associated with the avoidance of cancer screening [17]. In a survey of Japanese students, the percentage of students who thought cancer was scary decreased slightly as the students progressed from elementary to high school; however, the percentage of those who thought that cancer was preventable also decreased [18]. The number of those who were willing to undergo cancer screening when they were old enough to do so decreased as the school year progressed [18]. Although the cancer screening rate increases with age, the issue is how to increase the intention to undergo cancer screening, especially among young people.

The results of site-specific cancer screening among men showed that a family history of cancer and age were associated with screening for stomach, lung, and colorectal cancers. A U.S. study reported that people of older age had a higher screening rate for colorectal cancer [19], which is consistent with the results from this study. When those with and without a family history of stomach cancer were compared, the former had a higher cancer screening rate [20]. The study also showed an association between a higher income and stomach cancer screening. The results are consistent with previous studies showing that high-income individuals were more likely to receive stomach cancer screening than low-income individuals [20]. Previous studies from overseas found that age, educational level, marital status, and fear were not associated with cancer screening; however, family influence was associated with screening for cancer [21].

Japan’s population is aging, and screening is important to reduce cancer mortality. In the U.S., for many people whose level of education and income are low, they tend to not have health insurance, which led to a low colorectal cancer screening rate in one study [17]; however, in Japan, almost all citizens have health insurance. Nonetheless, as with physical examinations [22], it can be inferred that the factors related to social class have an impact on cancer screening.

Regarding site-specific cancer screening for women, the breast cancer screening rate (70.2%) was the highest among the cancer screenings in this study. This was close to the 75.6% previously reported by Hirai et al. [23]. This study also showed an association between a higher household income and cancer screening among women of child-rearing age consistent with the findings of previous studies [24]. In addition, prior studies have shown that regular cancer screening can be interrupted by pregnancy, childbirth, and breastfeeding [25].

For uterine cancer, when comparing women of the same generation, it has been reported that the consultation rate for women with children is higher than that for women without children in their early 20s, about the same in their late 20s, and lower in their 30s [26]. However, in this study, the odds ratios were significantly higher in women in their 30s, 40s, and 50s than in those in their 20s. The results of a previous study [27] also showed a positive correlation between age and the cancer screening rate. When considering the incidence of cancer among the adolescent and young adult generation, cervical cancer is the fifth-most common cancer among those in their 20s, while breast cancer and cervical cancer are the first and second-most common cancers, respectively, among those in their 30s [28]. In a previous study of college students, 93.0% and 11.5% of medical (nursing college) and nonmedical students, respectively, knew the cause of cervical cancer [29]. Moreover, the rate of undergoing cervical cancer screening among students was also significantly higher in the medical group [29]. In other words, adequate and proper knowledge may encourage screening.

In addition, in a previous study, significantly more participants who were married and had given birth underwent cancer screening [27,30]. However, uterine cancer screening is often included in the basic screening for pregnant women; therefore, 66.3% of the subjects in this study, most of whom had given birth, were screened. This rate was higher than the average screening rate in Japan.

Intervention acceptability was achieved by understanding the reasons why people of low socioeconomic status do not participate in cancer screening, targeting a low awareness of cancer screening and addressing inaccurate risk perceptions [31]. In Japan, each municipality sends information to those who are eligible, but if they are not interested, there is a high possibility that they will just throw it away. If the reasons why people do not participate in cancer screening can be clarified and information can be disseminated using SNS and messaging applications and reminders can be sent, it is presumed that interest in receiving cancer screening will increase.

In the U.S., breast and uterine cancer screening have been found to be associated with age and educational level [32]. Similarly, in this study, education was also associated with uterine cancer screening. The percentage of correct answers to questions assessing knowledge about cervical cancer screening was higher in participants who underwent screening than in those who did not [30]; therefore, it will be necessary to increase knowledge about uterine cancer and screening.

For the screening rates for stomach and colorectal cancers, a higher household income was associated with increased cancer screening. In a previous study, a higher household income, being employed, and being married were associated with a higher possibility of being screened for cancer [33], which is consistent with having a higher household income in the current study.

Considering that a high awareness of healthcare was also a motivating factor for undergoing cancer screening, educational opportunities for people to acquire knowledge about health and cancer will be necessary to improve the screening rate. In Sweden, cancer survival rates differ significantly with the educational level [11]. Hirai et al. reported an association between high health literacy and breast cancer screening [23]. Kawai et al. also clarified the relationship between health literacy and cervical cancer screening behavior [27]. In Japan, there are more opportunities that include cancer education in the school curriculum than before [34,35]. As a result, child-rearing generations may also have more opportunities to learn about cancer through their children. There is a need to improve health literacy through cancer education, and this survey shows that it is also important for the child-rearing generation.

## 5. Limitations

First, although access to cancer screening varies according to employment status, this study did not ask about employment status and, therefore, could not consider the ease of access to cancer screening. Second, we did not consider knowledge about cancer screening. Not only economic status but occupational status may also be important factors, and therefore, it may be necessary to provide opportunities for people to understand the importance of cancer knowledge and cancer screening [36]. Third, we were not able to ask about the factors that inhibit cancer screening or reasons for not wanting to or not being able to go for cancer screening. Finally, this study collected data from respondents’ answers, so a selection bias could not be ruled out that respondents are likely to be young and have a higher interest in health.

## 6. Conclusions

In this study, educational level, household income, age, and a family history of cancer were found to be associated with cancer screening uptake. Based on the results, cancer education would be required to improve the cancer screening uptake rate among the child-rearing generation, especially for those with low levels of education, low incomes, and no family history of cancer.

## Figures and Tables

**Table 1:** Participant demographic profile.

Parameter		*n*	%
Sex			
	Male	1228	51.0
	Female	1182	49.0
Age			
	20–29	25	1.0
	30–39	474	19.7
	40–49	1415	58.7
	50–59	454	18.8
	60–69	42	1.7
Living area			
	Hokkaido and Tohoku	267	11.1
	Kanto	851	35.3
	Hokuriku	96	4.0
	Chubu	337	14.0
	Kansai	393	16.3
	Chugoku	135	5.6
	Shikoku	67	2.8
	Kyushu	264	11.0
Child’s grade			
	Elementary 1–3	931	38.6
	Elementary 4–6	902	37.4
	Junior high school	847	35.1
	High school	762	31.6
Number of children			
	1	647	26.8
	2	1272	52.8
	3	412	17.1
	4 or more	79	3.3
Education			
	Junior high or high school	555	23.0
	Junior college or technical school	582	24.1
	University or graduate school	1273	52.8
Income			
	<4,000,000 yen	315	13.1
	≥4,000,000; <6,000,000 yen	705	29.3
	≥6,000,000; <8,000,000 yen	630	26.1
	≥8,000,000 yen	760	31.5
Cancer experience (multiple answer)			
	Own	94	3.9
	Partner	89	3.7
	Parent	800	33.2
	Grandparent	667	27.7
	Child	35	1.5
	Others	289	12.0
	None	907	37.6
Family member who died from cancer (multiple answer) *n* = 1886			
	Partner	12	0.6
	Parent	466	24.7
	Grandparent	577	30.6
	Child	6	0.3
	Others	160	8.5
	None	1353	71.7

**Table 2:** Cancer screening experiences.

Parameter	*n*	%	*n*	%	*n*	%
Cancer screening experience	Total (*n* = 2410)		Men (*n* = 1228)		Women (*n* = 1182)	
Yes	1381	57.3	503	41.0	878	74.3
No	1029	42.7	725	59.0	304	25.7
	Total aged > 40 years (*n* = 1911)		Men aged > 40 years (*n* = 1062)		Women aged > 40 years (*n* = 849)	
Cancer screening experience (>40s)						
Yes	1130	59.1	465	43.8	665	78.3
No	781	40.9	597	56.2	184	21.7
	Total (*n* = 2410)		Men aged > 40 years (*n* = 1062)		Women aged > 40 years (*n* = 849)	
Experience with cancer screening (multiple answer)						
Lung cancer	491	20.4	268	25.2	187	22.0
Stomach cancer	703	29.2	356	33.5	279	32.9
Colorectal cancer	708	29.4	331	31.2	317	37.3
Breast cancer	716	60.6	0	0.0	596	70.2
Uterine cancer *	784	66.3	0	0.0	784	66.3
Other cancer	120	5.0	78	7.3	21	1.8
Cancer screening experience (>40s)/Yes	*n* = 1130		*n* = 465		*n* = 665	
Income **						
<4,000,000 yen	162	11.7	38	8.2	78	11.7
≥4,000,000; <6,000,000 yen	401	29.0	114	24.5	199	29.9
≥6,000,000; <8,000,000 yen	345	25.0	116	24.9	167	25.1
≥8,000,000 yen	473	34.3	197	42.4	221	33.2
Education **						
Junior high or high school	297	21.5	75	16.1	155	23.3
Junior college or technical school	363	26.3	49	10.5	261	39.2
University or graduate school	721	52.2	341	73.3	249	37.4
Living area **						
Hokkaido and Tohoku	166	12.0	57	12.3	74	11.1
Kanto	492	35.6	157	33.8	247	37.1
Hokuriku	52	3.8	20	4.3	23	3.5
Chubu	184	13.3	55	11.8	96	14.4
Kansai	217	15.7	73	15.7	108	16.2
Chugoku	78	5.6	24	5.2	37	5.6
Shikoku	41	3.0	23	4.9	14	2.1
Kyushu	151	10.9	56	12.0	66	9.9

* Uterine cancer screening began at the age of 20. ** Only participants who had been screened for cancer.

**Table 3:** Factors associated with cancer screening experience.

Parameter	OR	95% CI	*p*
Sex			
Male	Ref.	----	----
Female	5.31	4.24–6.65	<0.001
Income			
<4,000,000 yen	Ref.	----	----
≥4,000,000; <6,000,000 yen	1.34	0.95–1.90	0.093
≥6,000,000; <8,000,000 yen	1.38	0.97–1.96	0.078
≥8,000,000 yen	1.79	1.26–2.54	0.001
Education			
Junior high or high school	Ref.	----	----
Junior college or technical school	1.10	0.81–1.49	0.539
University or graduate school	1.36	1.04–1.78	0.024
Family member with cancer			
No	Ref.	----	----
Yes	1.69	1.38–2.07	<0.001

95% CI (95% confidence interval).

**Table 4:** Factors associated with site-specific cancer screening experience in men.

		Lung Cancer			Stomach Cancer			Colorectal Cancer		
Parameter		OR	95% CI	*p*	OR	95% CI	*p*	OR	95% CI	*p*
Income										
<4,000,000 yen		Ref.	----	----	Ref.	----	----	Ref.	----	----
≥4,000,000; <6,000,000 yen		1.14	0.65–2.03	0.647	1.39	0.82–2.34	0.217	0.96	0.58–1.59	0.876
≥6,000,000; <8,000,000 yen		1.26	0.71–2.24	0.422	1.34	0.79–2.27	0.275	0.84	0.50–1.41	0.511
≥8,000,000 yen		1.69	0.97–2.94	0.062	1.71	1.02–2.85	0.041	1.24	0.76–2.04	0.385
Education										
Junior high or high school		Ref.	----	----	Ref.	----	----	Ref.	----	----
Junior college or technical school		1.02	0.59–1.74	0.956	0.81	0.49–1.32	0.394	0.85	0.51–1.40	0.517
University or graduate school		1.21	0.81–1.80	0.358	1.18	0.82–1.69	0.375	1.24	0.86–1.77	0.255
Family member with cancer										
No		Ref.	----	----	Ref.	----	----	Ref.	----	----
Yes		1.43	1.06–1.93	0.019	1.57	1.20–2.07	0.001	1.43	1.09–1.88	0.011
Age										
40s		Ref.	----	----	Ref.	----	----	Ref.	----	----
50s		1.89	1.41–2.53	<0.001	1.66	1.26–2.19	<0.001	1.26	0.95–1.67	0.107
60s		2.54	1.25–5.14	0.010	2.72	1.37–5.38	0.004	2.33	1.18–4.59	0.015

95% CI (95% confidence interval), adjusted for the living area.

**Table 5:** Factors associated with site-specific cancer screening experience in women.

Parameter	OR	95% CI	*p*	OR	95% CI	*p*	OR	95% CI	*p*	OR	95% CI	*p*
	Lung cancer			Stomach cancer			Colorectal cancer			Breast cancer		
Income												
<4,000,000 yen	Ref.	----	----	Ref.	----	----	Ref.	----	----	Ref.	----	----
≥4,000,000; <6,000,000 yen	1.87	1.00–3.50	0.490	2.33	1.34–4.08	0.003	1.92	1.16–3.20	0.012	1.27	0.80–2.01	0.307
≥6,000,000; <8,000,000 yen	1.83	0.96–3.49	0.067	2.57	1.45–4.56	0.001	1.95	1.15–3.31	0.014	1.65	1.01–2.70	0.046
≥8,000,000 yen	1.83	0.96–3.49	0.065	2.35	1.32–4.16	0.004	2.36	1.40–4.00	0.001	2.38	1.43–3.95	0.001
Education												
Junior high or high school	Ref.	----	----	Ref.	----	----	Ref.	----	----	Ref.	----	----
Junior college or technical school	1.39	0.88–2.18	0.158	1.09	0.74–1.62	0.656	0.98	0.68–1.43	0.925	1.24	0.85–1.80	0.267
University or graduate school	1.55	0.97–2.48	0.067	1.40	0.94–2.10	0.102	1.21	0.82–1.78	0.340	1.29	0.86–1.93	0.226
Family member with cancer												
No	Ref.	----	----	Ref.	----	----	Ref.	----	----	Ref.	----	----
Yes	1.53	1.06–2.20	0.024	1.70	1.23–2.34	0.001	1.77	1.30–2.41	0.001	1.69	1.24–2.30	0.001
Age												
40s	Ref.	----	----	Ref.	----	----	Ref.	----	----	Ref.	----	----
50s	1.07	0.65–1.76	0.799	1.57	1.02–2.41	0.041	1.07	0.70–1.64	0.076	0.94	0.59–1.49	0.775
60s	5.11	0.81–32.35	0.083	3.11	0.49–19.7	0.229	2.15	0.34–13.50	0.414	1.22	0.13–11.50	0.864

95% CI (95% confidence interval), adjusted for the living area.

**Table 6:** Factors associated with the uterine cancer screening experience in women.

Parameter	OR	95% CI	*p*
	Uterine cancer *		
Income			
<4,000,000 yen	Ref.	----	----
≥4,000,000; <6,000,000 yen	1.32	0.92–1.90	0.138
≥6,000,000; <8,000,000 yen	1.33	0.90–1.97	0.158
≥8,000,000 yen	1.79	1.18–2.70	0.006
Education			
Junior high or high school	Ref.	----	----
Junior college or technical school	1.29	0.94–1.77	0.110
University or graduate school	1.50	1.08–2.07	0.014
Family member with cancer			
No	Ref.	----	----
Yes	1.69	1.31–2.18	<0.001
Age			
20s	Ref.	----	----
30s	3.00	1.09–8.31	0.034
40s	4.56	1.67–12.47	0.003
50s	3.58	1.22–10.48	0.020
60s	6.28	0.55–71.43	0.139

* Uterine cancer screening begins at the age of 20 years in Japan. 95% CI (95% confidence interval), adjusted for the living area.

## Data Availability

The data presented in this study are available on request from the corresponding author.
